# An AIE-Active NIR Fluorescent Probe with Good Water Solubility for the Detection of Aβ_1–42_ Aggregates in Alzheimer’s Disease

**DOI:** 10.3390/molecules28135110

**Published:** 2023-06-29

**Authors:** Yan-Ming Ji, Min Hou, Wei Zhou, Zhang-Wei Ning, Yuan Zhang, Guo-Wen Xing

**Affiliations:** 1 Center of Safety Production and Testing Technology, China Academy of Safety Science and Technology, Beijing 100012, China; 2 College of Chemistry, Beijing Normal University, Beijing 100875, China; 3 Key Laboratory of Energy Conversion and Storage Materials, Beijing Normal University, Beijing 100875, China; 4 Key Laboratory of Radiopharmaceuticals, Ministry of Education, Beijing Normal University, Beijing 100875, China

**Keywords:** Alzheimer’s disease, AIE, Aβ, tetraphenylethylene, near-infrared imaging, amyloid, lactose, fluorescence

## Abstract

Alzheimer’s disease (AD), an amyloid-related disease, seriously endangers the health of elderly individuals. According to current research, its main pathogenic factor is the amyloid protein, which is a kind of fibrillar aggregate formed by noncovalent self-assembly of proteins. Based on the characteristics of aggregation-induced emission (AIE), a bislactosyl-decorated tetraphenylethylene (TPE) molecule **TMNL** (TPE + malononitrile + lactose), bearing two malononitrile substituents, was designed and synthesized in this work. The amphiphilic **TMNL** could self-assemble into fluorescent organic nanoparticles (FONs) with near-infrared (NIR) fluorescence emission in physiological PBS (phosphate buffered saline), achieving excellent fluorescent enhancement (47-fold) upon its combination with Aβ_1–42_ fibrils. **TMNL** was successfully applied to image Aβ_1–42_ plaques in the brain tissue of AD transgenic mice, and due to the AIE properties of **TMNL**, no additional rinsing process was necessary. It is believed that the probe reported in this work should be useful for the sensitive detection and accurate localization mapping of Aβ_1–42_ aggregates related to Alzheimer’s disease.

## 1. Introduction

Alzheimer’s disease, an incurable neurodegenerative disease, seriously endangers the physical and psychological health of elderly individuals [1,2,3]. One of the pathological features of Alzheimer’s disease is the abnormal deposition and accumulation of β-amyloid outside neurons in the cerebral cortex [4,5,6]. When the environment of the protein is changed, such as the temperature, pH, etc., or the protein is misfolded, its biological activity will decrease or even disappear, and the inactive protein aggregates, forming amyloids [7,8]. The most common types of β-amyloids in human bodies are Aβ_1–40_ and Aβ_1–42_, and Aβ_1–42_ polypeptides are more prone to aggregate and deposit into fibrillar aggregates [9,10,11,12].

Developing sensitive and efficient tools for the accurate sensing of Aβ polypeptides is of great importance to the diagnosis and intervention of AD in its early stage. At present, most reported fluorescent probes used for Aβ imaging, such as Thioflavin T (ThT), Thioflavin S (ThS), BODIPYs, and oxazines [13,14,15], lead to serious self-fluorescence quenching due to aggregation at the Aβ binding site, and the rinse process also needs to be repeatedly performed during real-time imaging to overcome the disadvantages of the aggregation-caused quenching (ACQ) effect. In addition, ThT and Congo red (CR) fluorophores with short emission wavelengths and small Stokes shifts are not suitable for imaging in vivo [16,17]. Several fluorescent probes with D-π-A structures and the intramolecular charge transfer (ICT) effect have been developed to extend the conjugate system; however, the fluorescent defects of the molecules themselves still limit their wide application to achieve accurate imaging information for mapping Aβ plaques [18,19,20].

According to the research and report of Tang’s group [21], AIE was a preferential strategy to identify protein fibrillar formation [16]. In contrast with the ACQ fluorophores, fluorophores with AIE properties were exactly able to compensate for their deficiency, particularly the light-up characteristic during the aggregation process [21]. In addition, low fluorescence background, high sensitivity, and good resistance to photobleaching are also advantages of AIE-type luminogens. AIE-luminogen (AIEgen) itself, in dilute solution, exhibited weak fluorescence due to intramolecular thermal motion, but when it was combined with the β-sheet of amyloid due to hydrophobic interactions, the fluorescence was greatly enhanced under the restriction of intramolecular motion (RIM) effect [22,23,24], enabling fluorescence imaging of amyloids, such as Aβ_1–42_ fibrils [16,21]. However, the initial aggregation of AIE fluorescent probes with poor water solubility led to a stronger “false-positive” AIE signal before binding to Aβ aggregates. To date, a number of fluorescent probes have been developed to detect Aβ amyloid [14,25,26,27,28,29,30,31,32,33,34,35], but water-soluble NIR fluorophores with AIE properties have rarely been reported [36,37,38,39,40].

## 2. Results and Discussion

According to the reported literature [20], the malononitrile substituent could be used as an acceptor in D-π-A type NIR fluorophores for the detection of Aβ fibrils, and the π bridge could enhance the fluorescence emission of the probe and increase its redshift. In addition, lactose is a highly biocompatible and water-soluble substance, and it is a good choice for use as the hydrophilic unit.

**Scheme 1:**
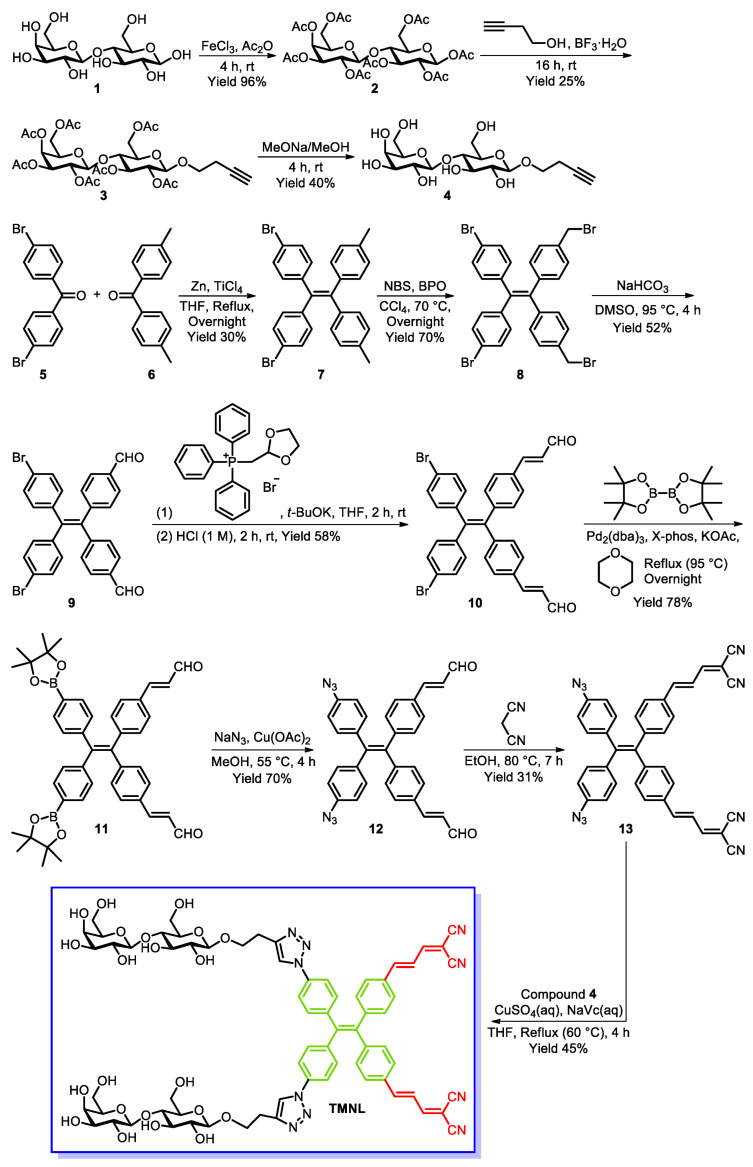
The synthesis route of **TMNL**.

Herein, we designed and synthesized a bislactosyl-decorated tetraphenylethylene (TPE) molecule, **TMNL** (Scheme 1), with typical AIE fluorescence characteristics, which contained two malononitrile substituents in the molecule. The amphiphilic **TMNL** could self-assemble into fluorescent organic nanoparticles (FONs) with NIR fluorescence emission in PBS buffer solution (pH 7.4) and could achieve excellent no-rinsing fluorescence imaging of Aβ_1–42_ fibrils through the combination of malononitrile groups with Aβ_1–42_ fibrils. **TMNL** was bestowed on the following extraordinary features. (i) The water-soluble lactose units would increase the water solubility and biocompatibility of TPE. (ii) The AIE-active TPE unit would overcome the ACQ effect of traditional fluorophores. (iii) The malononitrile substituent with an electron-withdrawing effect could extend the conjugated system of TPE, which redshifted the emission wavelength to the NIR region. Compared to the reported AIE-type NIR fluorescence probes for the detection of the Aβ amyloid (Tables S1 and S3), **TMNL** was the first amphiphilic and water-soluble AIE-active NIR fluorescent probe with a large Stokes shift for the detection of Aβ_1–42_ and high-fidelity in situ mapping of Aβ_1–42_ plaques.

### 2.1. Synthesis

The details of the synthetic routes of **TMNL** are shown in Scheme 1. Briefly, the tetraphenylethene derivative, Compound **9**, was synthesized with **5** and **6** as starting materials by McMurry coupling reaction, radical substitution reaction, and Kornblum oxidation reaction. The double bond was introduced to Compound **9** by the Witting reaction and then coupled to malononitrile by the Knoevenagel reaction to afford **13**. Finally, **TMNL** was obtained by the click reaction of lactose derivative **4** and TPE derivative **13**. The chemical structures of **TMNL** and the synthetic intermediates were characterized by ^1^H NMR, ^13^C NMR, and HRMS (Figures S1–S5).

### 2.2. Photophysical Characterization of ***TMNL***

**Figure 1:**
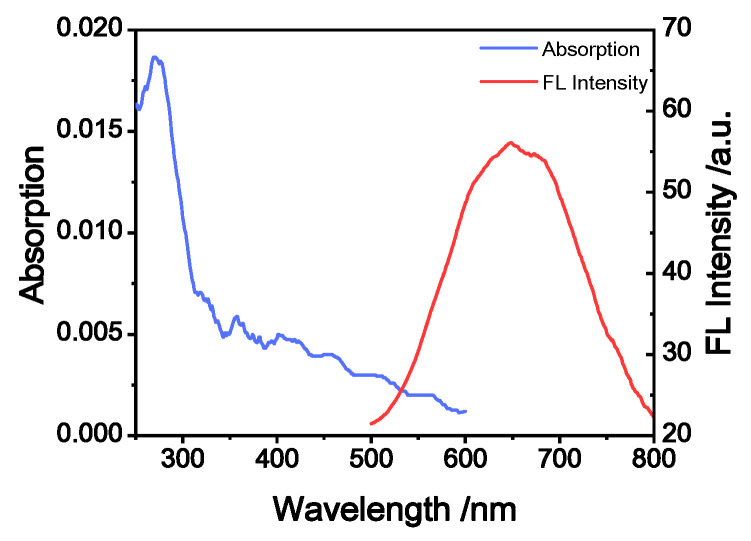
The UV absorption spectra and fluorescence emission spectra of **TMNL**.

We first investigated the photophysical properties of **TMNL** under physiological conditions (PBS buffer, pH 7.4). **TMNL** showed weak absorption at approximately 320–380 nm, and the maximum emission wavelength was 645 nm (Figure 1). The large Stokes shift of approximately 285 nm could reduce the self-absorption effect and make **TMNL** have a strong ability to resist background interference [41,42].

**Figure 2:**
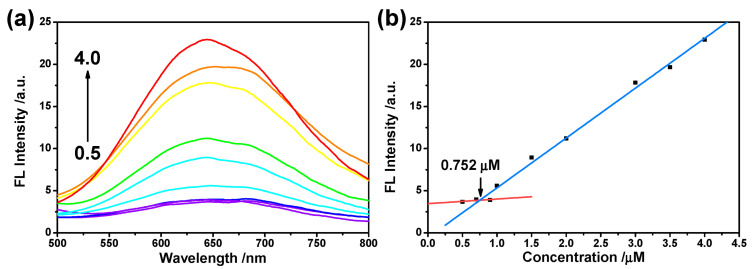
(**a**) The fluorescence spectra of different concentrations (μM) of **TMNL** in PBS buffer solution (pH 7.4, 10 mM). (**b**) Scatter plot of fluorescence intensity at 645 nm of different concentrations of **TMNL** in PBS buffer solution (pH 7.4, 10 mM). λ_ex_ = 360 nm.

Initial background minimization and fidelity signal amplification of **TMNL** were essential for ultrasensitive and accurate detection of Aβ_1–42_ fibrils; therefore, it was necessary to choose a suitable detection concentration to avoid a “false-positive” AIE signal. The fluorescence spectra of **TMNL** itself as a function of concentration were first measured. As shown in Figure 2, when the concentration of **TMNL** was less than 0.752 μM, the fluorescence intensity in PBS buffer solution (pH 7.4, 10 mM) did not change obviously with increasing concentration. Therefore, the critical micelle concentration (CMC) was measured at 0.752 μM, and **TMNL** could self-assemble to form FONs when the concentration exceeded 0.752 μM. To make the fluorescence intensity of **TMNL** as low as possible before binding to Aβ_1–42_ fibrils, 1 μM was selected as the test concentration.

### 2.3. The Performance of Aβ_1–42_ Fibrils Detection

**Figure 3:**
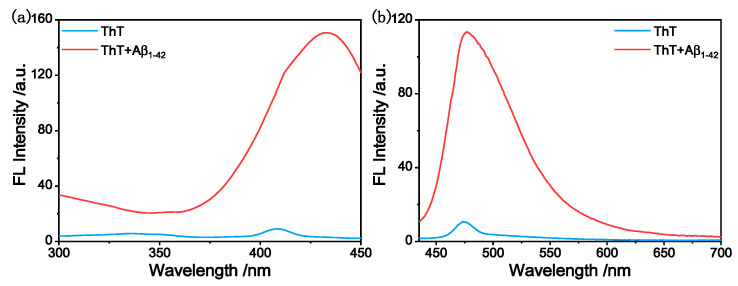
Fluorescence spectra of the excitation (**a**) and emission (**b**) of ThT (1 μM)-detecting Aβ_1–42_ (80 μg·mL^−1^) fibrils in PBS buffer solution (pH 7.4, 10 mM). λ_ex_ = 410 nm, λ_em_ = 470 nm.

The binding properties of **TMNL** to Aβ_1–42_ fibrils were mainly characterized by fluorescence spectroscopy in PBS buffer solution (pH 7.4, 10 mM). The Aβ_1–42_ species included monomers, oligomers, and aggregates, and different aggregation degrees could have a critical influence on the change in fluorescence intensity [39]. The Aβ_1–42_ fibrils were prepared from the Aβ_1–42_ peptide by coincubation in PBS buffer solution (pH 7.4, 10 mM) at 37 °C for seven days (please refer to Section 3.3 for the detailed steps). ThT is generally used as an authoritative standard probe for the detection of the aggregation state of amyloid [37,43,44,45,46]. As shown in Figure 3, upon coincubation with treated Aβ_1–42_ fibrils, the fluorescence intensity of the excitation spectra and the emission spectra sharply enhanced, accompanied by a redshift, which indicated that the Aβ_1–42_ protein was in a good aggregation state and could be used for the following detection.

**Figure 4:**
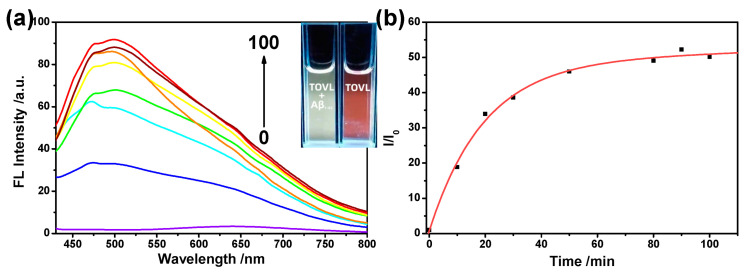
(**a**) Fluorescence spectra of the interaction of **TMNL** with Aβ_1–42_ fibrils after different coincubation times in PBS buffer solution (pH 7.4, 10 mM). Insert: photograph of **TMNL** after complete interaction with Aβ_1–42_ fibrils (**left**) and in the absence of Aβ_1–42_ fibrils (**right**) in quartz cuvettes under 365 nm UV light. (**b**) Scatter plot of the relative fluorescence intensity (I/I_0_) at 496 nm of the different coincubation times of **TMNL** and Aβ_1–42_ fibrils in PBS buffer solution (pH 7.4, 10 mM). I and I_0_ represent the fluorescence intensity in the presence and absence of Aβ_1–42_ fibrils, respectively. [**TMNL**] = 1 μM, [Aβ_1–42_] = 80 μg·mL^−1^, λ_ex_ = 360 nm.

**Figure 5:**
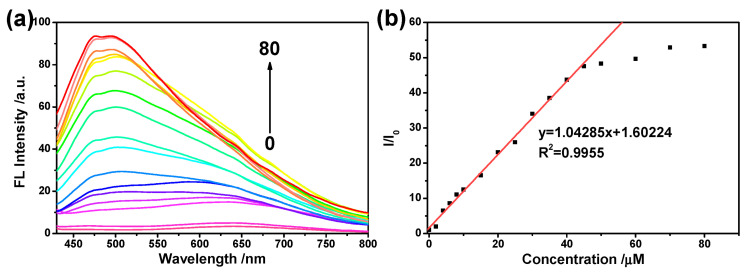
(**a**) Fluorescence spectra of the interaction of **TMNL** with different concentrations of Aβ_1–42_ fibrils (μg·mL^−1^) in PBS buffer solution (pH 7.4, 10 mM). (**b**) Scatter plot of the relative fluorescence intensity (I/I_0_) at 496 nm of the interaction of **TMNL** and different concentrations of Aβ_1–42_ fibrils (μg·mL^−1^) in PBS buffer solution (pH 7.4, 10 mM). I and I_0_ represent the fluorescence intensity in the presence and absence of Aβ_1–42_ fibrils, respectively. [**TMNL**] = 1 μM, λ_ex_ = 360 nm.

**Figure 6:**
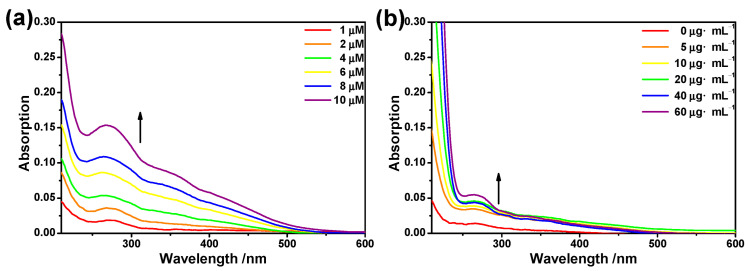
(**a**) The UV absorption spectra of different concentrations of **TMNL** in PBS buffer solution (pH 7.4, 10 mM). (**b**) UV absorption spectra of the interaction of **TMNL** (1 μM) with different concentrations of Aβ_1–42_ fibrils (μg·mL^−1^) in PBS buffer solution (pH 7.4, 10 mM).

To clarify the saturation time of the interaction between **TMNL** and Aβ_1–42_ fibrils, the “Time-Fluorescence Intensity” experiment was performed in PBS buffer solution (pH 7.4, 10 mM). After coincubation with Aβ_1–42_ fibrils, the fluorescence emission intensity at 645 nm was slightly enhanced, but a very obvious emission peak appeared at 496 nm and increased rapidly with longer coincubation time, which can be attributed to the RIM effect by the binding of **TMNL** with the Aβ_1–42_ fibrils. The fluorescence intensity at 496 nm became constant within 60 min, and the color change in the solution before and after the addition of Aβ_1–42_ fibrils could be clearly distinguished with the naked eye (from red to yellow) under UV illumination at 365 nm (Figure 4, insert). When the coincubation time was fixed at 60 min, the fluorescence intensity of **TMNL** at approximately 496 nm increased gradually and finally tended to flatten with increasing Aβ_1–42_ fibril concentration. The increased fluorescence intensity was up to 47-fold (Figure 5). Moreover, the linear relationship between the Aβ_1–42_ fibril concentration and the fluorescence intensity of **TMNL** at 496 nm was obtained in the concentration range of 0–45 μg·mL^−1^ (R^2^ = 0.9955, Figure 5), indicating that the concentration of Aβ_1–42_ fibrils can be quantitatively estimated by **TMNL**. Then, the UV absorption spectra of **TMNL** in the presence and absence of Aβ_1–42_ fibrils were also determined. As shown in Figure 6a, the absorption band at 265–360 nm gradually increased with increasing concentrations of Aβ_1–42_ fibrils, which also indicated that the binding of **TMNL** and Aβ_1–42_ fibrils changed the conjugation system of the **TMNL** molecule. These results suggested that **TMNL** could act as an efficient and sensitive tool for the detection of Aβ_1–42_ fibrils.

### 2.4. Selectivity Study

**Figure 7:**
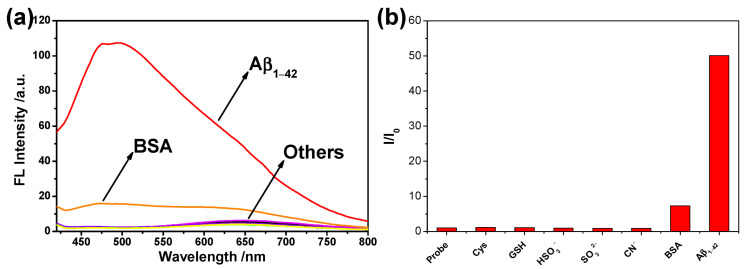
(**a**) Fluorescence spectra of the interaction of **TMNL** with different interfering substances and Aβ_1–42_ fibrils in PBS buffer solution (pH 7.4, 10 mM). (**b**) The histogram of relative fluorescence intensity (I/I_0_) at 496 nm of the interaction of **TMNL** with different interfering substances and Aβ_1–42_ fibrils in PBS buffer solution (pH 7.4, 10 mM). I represents the fluorescence intensity in the presence of the interfering substances or Aβ_1–42_ fibrils, and I_0_ represents the fluorescence intensity in the presence of **TMNL** alone. [Cys] = [GSH] = [HSO_3_^−^] = [SO_3_^2−^] = [CN^−^] = 100 μM, [BSA] = [Aβ_1–42_] = 80 μg·mL^−1^; [**TMNL**] = 1 μM; λ_ex_ = 360 nm.

We also screened a series of sulfur-containing substances to verify the selectivity of **TMNL** for Aβ_1–42_ fibrils, including L(+)-cysteine (Cys), glutathione (GSH), HSO_3_^−^, SO_3_^2−^, and BSA. **TMNL** was incubated with various high-concentration interfering substances. As shown in Figure 7, the fluorescence intensity of **TMNL** was nearly unchanged in the presence of other sulfur-containing compounds. Although BSA could produce a weak fluorescence response, it was far lower than the 47-fold increase produced by Aβ_1–42_ fibrils, which suggested that **TMNL** could be used for highly selective detection of Aβ_1–42_ fibrils under complex physiological conditions. Compared to the reported probes (Table S2), **TMNL** had no inferior selectivity for Aβ_1–42_ fibrils.

### 2.5. The Dissociation Constant Study

**Figure 8:**
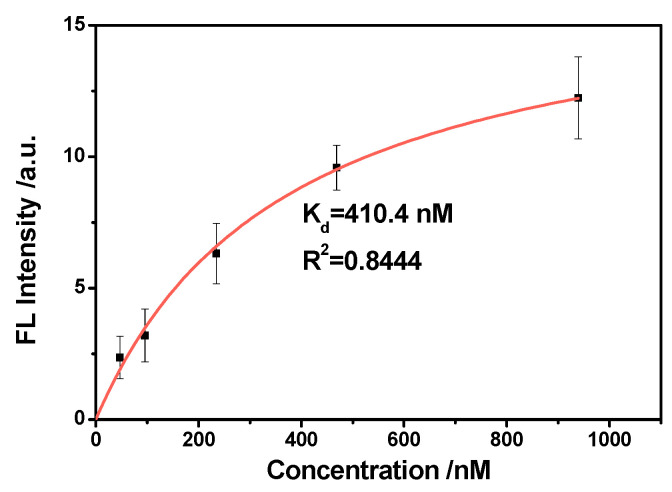
Saturation binding curves of different concentrations of **TMNL** interacting with Aβ_1–42_ fibrils. [Aβ_1–42_] = 80 μg·mL^−1^; λ_ex_ = 360 nm.

Binding affinity was a crucial factor for **TMNL** to efficiently trace the Aβ_1–42_ fibrils, and a saturation binding experiment was performed to quantitatively evaluate the binding ability of **TMNL** to Aβ_1–42_ fibrils. The fluorescence intensity was measured by incubating Aβ_1–42_ fibrils with different concentrations of **TMNL**, as shown in Figure 8. The dissociation constant K_d_ in this process was calculated to be 410.4 nM, which indicated that **TMNL** was a good substrate for Aβ_1–42_ fibrils and could be well applied to Aβ_1–42_ fibril detection. Compared to the reported AIE-type NIR fluorescence probes for the detection of Aβ amyloid (Tables S1 and S3), **TMNL** had a moderate binding affinity.

### 2.6. Appearance Observations

**Figure 9:**
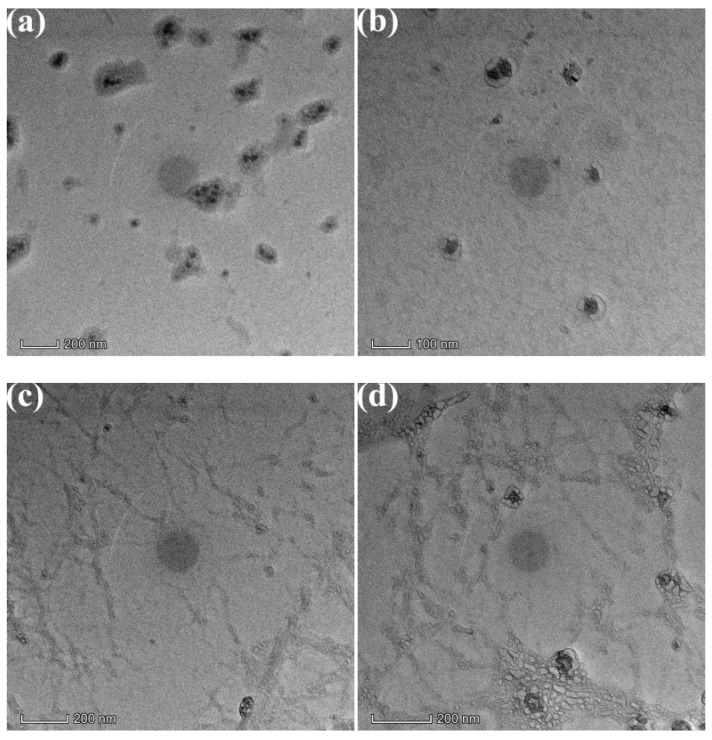
(**a**,**b**) TEM images of **TMNL** in PBS buffer solution (pH 7.4, 10 mM). (**c**,**d**) TEM images of **TMNL** after binding to Aβ_1–42_ fibrils in PBS buffer solution (pH 7.4, 10 mM). [**TMNL**] = 1 μM; [Aβ_1–42_] = 80 μg·mL^−1^.

To intuitively clarify the binding appearance of **TMNL** with Aβ_1–42_ fibrils, transmission electron microscopy (TEM) images of **TMNL** binding to Aβ_1–42_ fibrils were obtained. As shown in Figure 9a,b, **TMNL** could self-assemble into small spherical nanostructures 30–100 nm in diameter. After coincubation with Aβ_1–42_ fibrils in PBS buffer solution (pH 7.4, 10 mM) for 60 min, as shown in Figure 9c,d, **TMNL** aggregates were attached to the intertwined Aβ_1–42_ fibrils, which indicated that **TMNL** and Aβ_1–42_ fibrils could combine to make TPE aggregate and to emit strong fluorescence.

### 2.7. In Vitro Mapping with High-Fidelity Aβ_1–42_ Plaque Information

**Figure 10:**
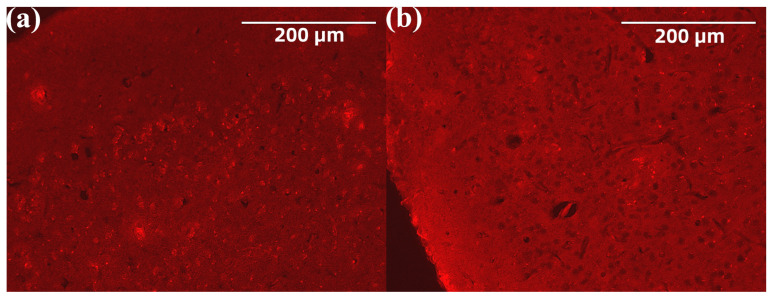
(**a**,**b**) Fluorescence staining images of **TMNL** in brain tissue sections of AD transgenic mice.

To evaluate the performance of **TMNL** in bioimaging-related fields, **TMNL** was applied to stain Aβ_1–42_ plaques in the brain tissue of AD transgenic mice (App, 13 months old). As shown in Figure 10, **TMNL** bound to and stained Aβ_1–42_ plaques in the brain tissue of AD transgenic mice, as observed by an Axio Observer Z1 microscope. Due to the AIE properties of **TMNL**, no additional rinsing or processing was necessary. These results preliminarily indicated that **TMNL** had a good affinity for Aβ_1–42_ plaques in biological tissues, which was suitable and convenient for further clinical application.

## 3. Materials and Methods

### 3.1. Materials and Instrumentations

All chemicals and solvents were purchased from commercial suppliers and used without further purification, unless otherwise noted. The Aβ_1–42_ polypeptide was obtained from GL Biochem (Shanghai, China) Co., Ltd. Bovine serum albumin (BSA) was purchased from Innochem (Beijing, China) Technology Co., Ltd. PBS buffer (pH 7.2–7.4, 0.01 M) was purchased from Beijing Solarbio Science & Technology Co., Ltd., Beijing, China. All anaerobic reactions were performed under a dry N_2_ atmosphere. All reactions were monitored by thin-layer chromatography (TLC) on T-HSGF10025025 normal-phase silica gel glass plates or 60 RP-18 F_254_s reversed-phase silica gel glass plates and revealed with UV light (254 nm or 365 nm) or EtOH-H_2_SO_4_ (7%) solution. Flash column chromatography was performed on 200–300 mesh silica gel. Reversed-phase chromatography was performed on SiliaSphere C18 (50 μm, 120 Å). Molecular exclusion chromatography was performed on Bio-Gel^®^ P-2 Media (45–90 μm). ^1^H and ^13^C NMR spectra were recorded on a Bruker Avance III spectrometer (400 MHz) or JNM-ECZR spectrometer (400 and 600 MHz) with tetramethylsilane (TMS, δ = 0 ppm) as an internal standard. The residual peaks of the solvent were chloroform-d at 7.26 ppm (^1^H) and 77.16 ppm (^13^C), methanol-d_4_ at 3.31 ppm (^1^H) and 49.00 ppm (^13^C), and DMSO-d_6_ at 2.50 ppm (^1^H) and 39.52 ppm (^13^C). High-resolution mass spectra were recorded on a Bruker micrOTOF-QII mass spectrometer (ESI) or Bruker autoflex speed LRF (MALDI-TOF). Photoluminescence (PL) spectra were recorded on a FS5 spectrofluorometer (Edinburgh Instruments Ltd., Edinburgh, UK). Transmission electron microscopy (TEM) images were recorded on a FEI Talos F200S (Thermo Fisher Scientific, Waltham, USA). Distilled water was used throughout the test experiments.

### 3.2. Preparation of AD Transgenic Mouse Brain Tissue Paraffin Sections

Brain tissue paraffin sections of AD transgenic mice (App, 13 months old) were obtained from the Institute of Laboratory Animals Science, CAMS & PUMC (Beijing, China). Pretreatment of the brain tissue paraffin sections of AD transgenic mice (App, 13 months old) included soaking the sections in dimethylbenzene solution for 5 min to dewax, rinsing with ethanol and secondary water, and finally air-drying for further use. The brain tissue paraffin section images were observed on an Axio Observer Z1 microscope (Carl Zeiss AG, Oberkochen, Germany).

### 3.3. Preparation of Aβ_1–42_ Fibrils

Lyophilized 2.5 mg Aβ_1–42_ polypeptide powder was dissolved in 1.25 mL of PBS buffer solution (pH 7.4, 10 mM), and a small amount of NH_3_ was added to fully dissolve Aβ_1–42_, obtaining a 2 mg·mL^−1^ Aβ_1–42_ stock solution. Then, the 2 mg·mL^−1^ Aβ_1–42_ stock solution was diluted in PBS buffer solution (pH 7.4, 10 mM) to obtain a 200 μg·mL^−1^ protein solution and incubated at 37 °C for 7 days at 120 r·min^−1^ in a constant temperature oscillator to obtain an Aβ_1–42_ aggregate solution.

### 3.4. Coincubation Preparation Process of TMNL with Aβ_1–42_ Fibrils

The **TMNL** was mixed with Aβ_1–42_ aggregate solution and then coincubated at 37 °C for 1 h at 120 r·min^−1^ in a constant temperature oscillator for further testing.

### 3.5. Synthesis of Compound ***4***

Compounds **1** to **3** were synthesized according to the methods reported in a previous article [47].

Compound **4**: Compound **3** (0.1 g, 0.054 mmol) was dissolved in dry methanol (5.42 mL), and a solution of MeONa/MeOH (5.4 M, 19 μL) was added with a microsyringe, and then the mixture was stirred for 4 h at room temperature. The reaction was neutralized with an IR-120 hydrogen ion resin to adjust the pH of the mixture to approximately 7. Then, the mixture was filtered, and the solvent was evaporated. The crude compound was purified by Bio-Gel^®^ P-2 gel (pure H_2_O) to obtain Compound **4** (41 mg, 40% yield). ^1^H NMR (400 MHz, CD_3_OD), δ (ppm): 4.82 (d, *J* = 3.9 Hz, 1H), 4.39–4.32 (m, 2H), 3.96–3.67 (m, 14H), 3.61–3.43 (m, 9H), 2.52 (td, *J* = 7.3, 2.7 Hz, 3H), 2.28 (t, *J* = 2.6 Hz, 1H).

### 3.6. Synthesis of Compound ***13***

Compounds **5** to **12** were synthesized according to the methods reported in a previous article [47].

Compound **13**: Compound **12** (50 mg, 0.096 mmol), malononitrile (51 mg, 0.77 mmol), and ethanol (4 mL) were added in a round bottom flask in that order. The mixture was then stirred at 80 °C until the reactant disappeared. After the reaction was accomplished, the reaction was cooled to room temperature, and the solvent was evaporated. The residue was purified by column chromatography (Hexane:EtOAc = 3:1, *v*/*v*) to obtain Compound **13** (18 mg, 31% yield). ^1^H NMR (400 MHz, CDCl_3_), δ (ppm): 7.57 (t, *J* = 5.4 Hz, 1H), 7.38 (d, *J* = 8.2 Hz, 2H), 7.19 (d, *J* = 5.1 Hz, 2H), 7.07 (d, *J* = 8.2 Hz, 2H), 6.99 (d, *J* = 8.4 Hz, 3H), 6.81 (d, *J* = 8.5 Hz, 2H); ^13^C NMR (100 MHz, CDCl_3_), δ (ppm): 159.71, 149.47, 147.02, 142.66, 139.45, 139.11, 139.03, 132.78, 132.64, 132.25, 128.75, 122.47, 118.80, 113.53, 111.72, 82.85; MS (MALDI-TOF): *m*/*z* Calcd for C_38_H_22_N_10_ [M − N_2_ + H]^+^ 591.205, found: 591.292.

### 3.7. Synthesis of Compound ***TMNL***

**TMNL**: Compound **13** (0.42 g, 0.11 mmol) and Compound **4** (30 mg, 0.049 mmol) were added to a double neck bottle and dissolved in THF (3 mL) under N_2_. Then, sodium ascorbate (0.019 M, 1 mL, aq.) and CuSO_4_ (0.096 M, 1 mL, aq.) were added successively and stirred at 60 °C for 4 h. Then, the reaction was cooled to room temperature, extracted with DCM, washed with saturated aqueous sodium chloride, and dried over Na_2_SO_4_. After filtration, the solvent of the mixture was evaporated. The crude product was purified by Bio-Gel P-2 gel (pure H_2_O) to obtain **TMNL** (0.14 g, 45% yield). ^1^H NMR (400 MHz, DMSO-d_6_), δ (ppm): 8.60 (s), 7.71–7.61 (m), 7.24–7.01 (m), 5.28 (s), 5.04 (s), 4.74 (s), 4.66 (s), 4.59 (s), 4.55–4.51 (m), 4.47 (s), 4.26 (d, *J* = 7.9 Hz), 4.16 (d, *J* = 6.6 Hz), 4.01 (s), 3.74–3.66 (m), 3.58 (s), 3.51–3.39 (m), 3.02 (s), 2.93 (s); HRMS (ESI): *m*/*z* Calcd for C_70_H_75_N_10_O_22_ [M + H]^+^ 1407.5057, found: 1407.5060.

## 4. Conclusions

In this work, an AIE-active water-soluble and near-infrared fluorescent luminogen, **TMNL**, was successfully designed, synthesized, and well characterized; it contained hydrophilic lactose units, hydrophobic malononitrile, and a TPE derivative moiety. **TMNL** could self-assemble into fluorescent organic nanoparticles in aqueous solution and was used for the detection of Aβ_1–42_ fibrils and the NIR imaging of Aβ_1–42_ plaques sensitively and selectively. After coincubation with Aβ_1–42_ fibrils, the fluorescence intensity of **TMNL** at 496 nm increased up to 47-fold, and it also had excellent selectivity for Aβ_1–42_ fibrils. The K_d_ value (410.4 nm) indicated the good affinity between **TMNL** and Aβ_1–42_ fibrils, which could also be observed by TEM. **TMNL** could be utilized for the imaging of Aβ_1–42_ plaques in brain tissue accurately and conveniently, which could be an alternative to commercial probes. Although **TMNL** still had some characteristics to be further improved, such as complex preparation, this work was expected to facilitate relevant studies on Alzheimer’s disease.

## Data Availability

The data that support the findings of this study are available from the corresponding author upon reasonable request.

## Supplementary Materials

The following supporting information can be downloaded at: https://www.mdpi.com/article/10.3390/molecules28135110/s1, Table S1: AIE-type fluorescent probes for the detection of Aβ amyloid; Table S2: The selectivity comparison of AIE-type fluorescent probes for the detection of Aβ amyloid; Table S3: The molecular structures of the corresponding probes in Tables S1 and S2; Figure S1: ^1^H NMR spectra of compound **4**; Figure S2: ^1^H NMR spectra of compound **13**; Figure S3: ^13^C NMR spectra of compound **13**; Figure S4: ^1^H NMR spectra of **TMNL**; Figure S5: HRMS spectra of **TMNL**.

## Sample Availability

Samples of the compounds are available from the authors.
