# Supplementary material for: An AIE-Active NIR Fluorescent Probe with Good Water Solubility for the Detection of Aβ1–42 Aggregates in Alzheimer’s Disease
Source: Molecules. 2023 Jun 29;28(13):5110. doi: 10.3390/molecules28135110 (PMC10343367; doi:10.3390/molecules28135110)
Supplement: Supplementary file 1 [file molecules-28-05110-s001.zip › molecules-2441686-supplementary.pdf]

1 Experimental Procedures

Table S1. AIE-type fluorescent probes for the detection of Aβ amyloid.

Probe name	Type	Maximum absorption / emission wavelength (nm)	Water-solubility	Emission enhancement (Folds) [1]	Amyloid	K_d (nM) [2]	Mapping of Aβ plaque [3]	Reference
Cur-N-BF_2	*Light-up*	470 / 572	PBS buffer solution	-	$Aβ_{1-42}$ fibrils	-	-	[1]
QM-FN-SO_3	*Off-on*	500 / 720	PBS buffer solution	50	$Aβ_{42}$ fibrils	170	in situ brain sections	[2]
FB	*Turn-on*	565 / 605	PBS buffer solution	20	$Aβ_{1-42}$ fibrils	47.91	brain sections	[3]
TM-1	*Turn-on*	500 / 680	1% DMSO in PBS buffer solution	106	Aβ aggregates	35	brain sections	[4]
TM-2		430 /650		96		92		
**TMNL**	*Turn-on*	360 / 645	PBS buffer solution	47	$Aβ_{1-42}$ fibrils	410.4	in situ brain sections	This probe

[1] "-" meant not mentioned.

[2] "-" meant not measured.

[3] "-" meant not mentioned.

Table S2. The selectivity comparison of AIE-type fluorescent probes for the detection of Aβ amyloid.

Probe name	Graph	Selectivity	Reference
Cur-N-BF_2	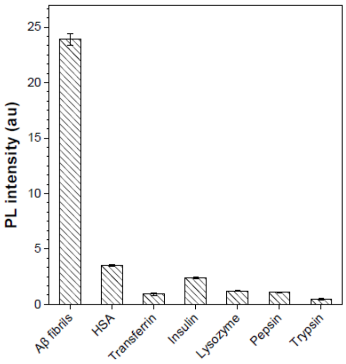	$A\beta_{1-42}$ fibrils	[1]
QM-FN-SO_3	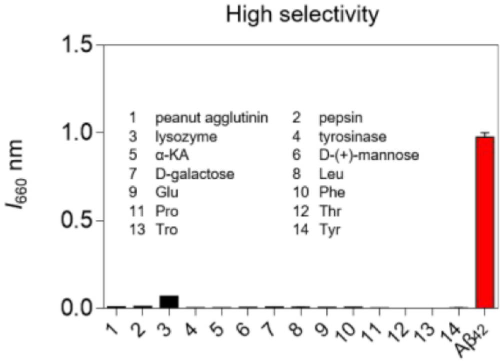	$A\beta_{42}$ fibrils	[2]
FB	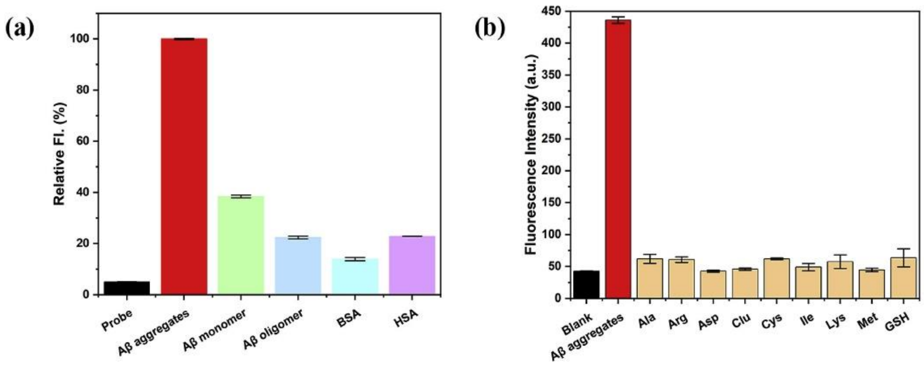	$A\beta_{1-42}$ fibrils	[3]
TM-1<br>TM-2	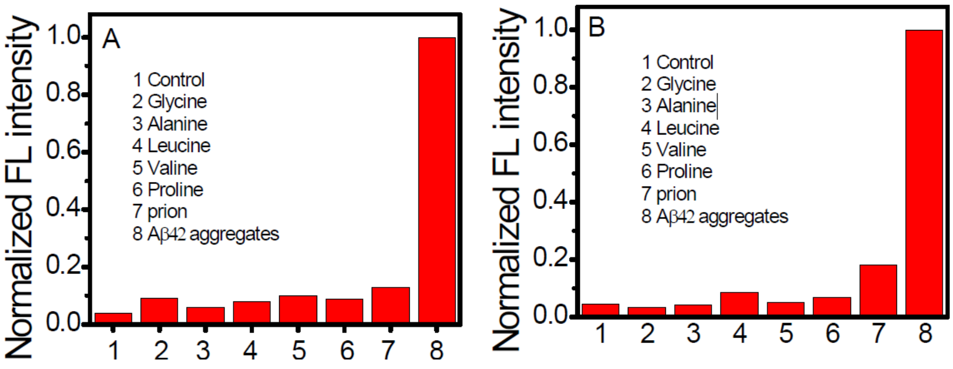	Aβ aggregates	[4]

Table S3. The molecular structures of the corresponding probes in Table S1-S2.

Probe name	Structure
Cur-N-BF$_2$	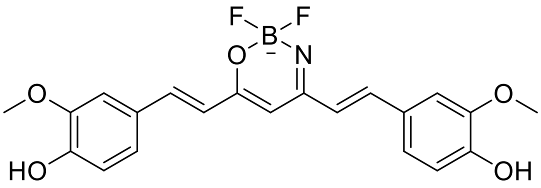
QM-FN-SO$_3$	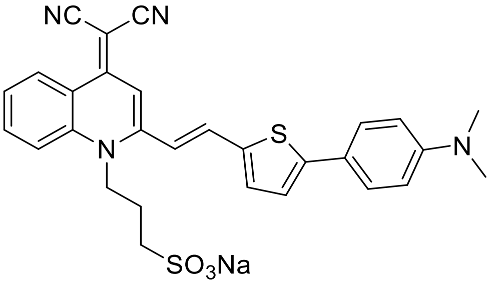
FB	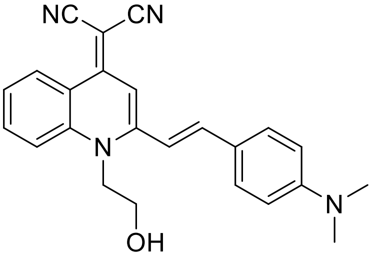
TM-1	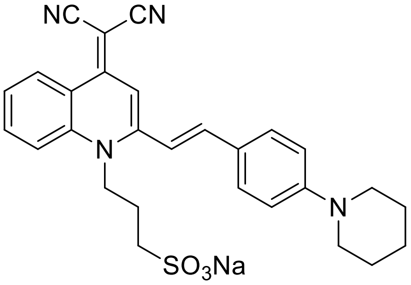
TM-2	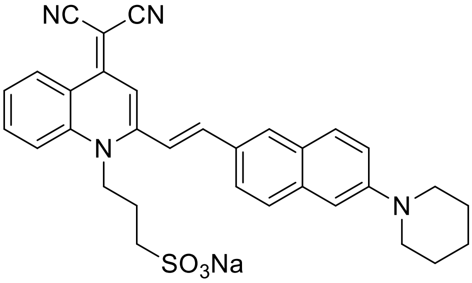

2 ^{1}H NMR, ^{13}C NMR and HRMS Spectrum of New Compounds

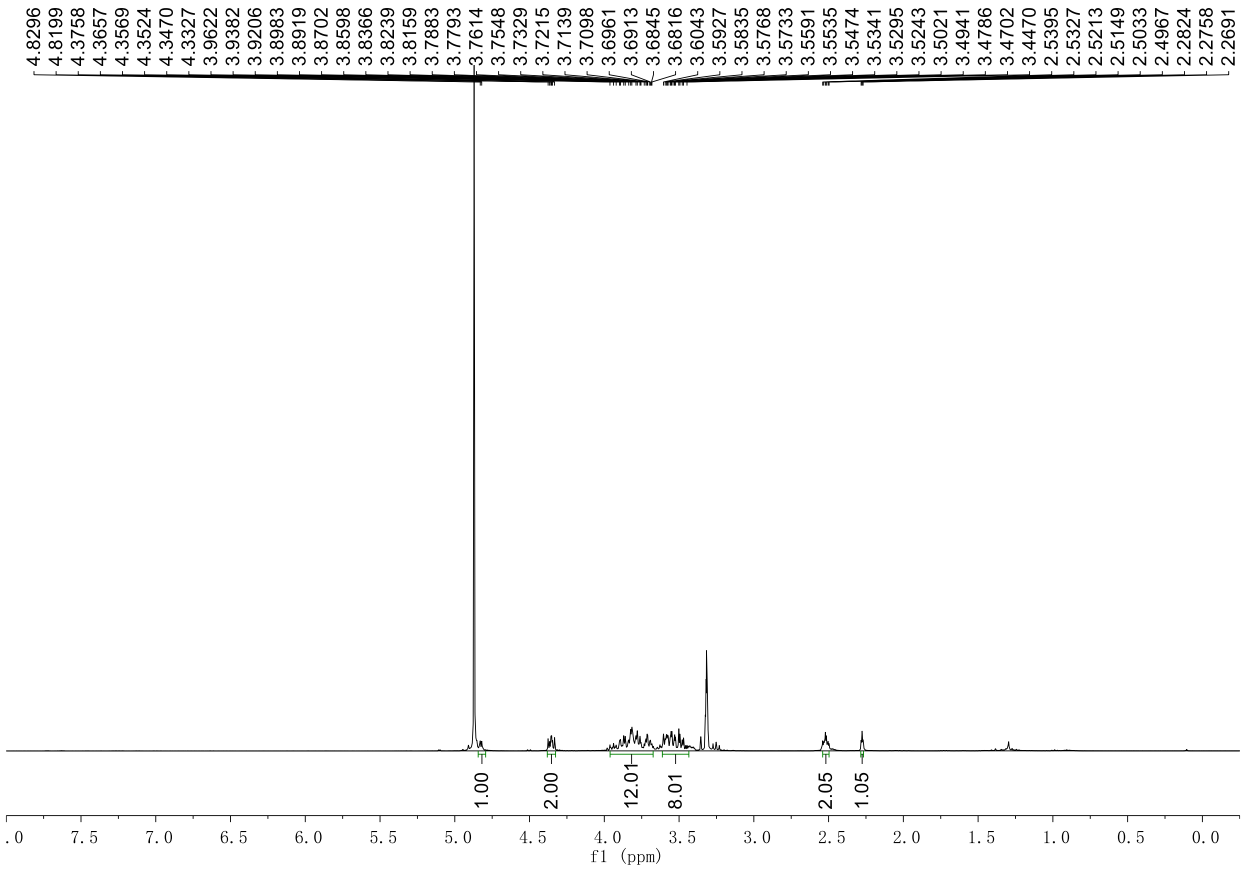

Figure S1. ^{1}H NMR spectra of compound **4**.

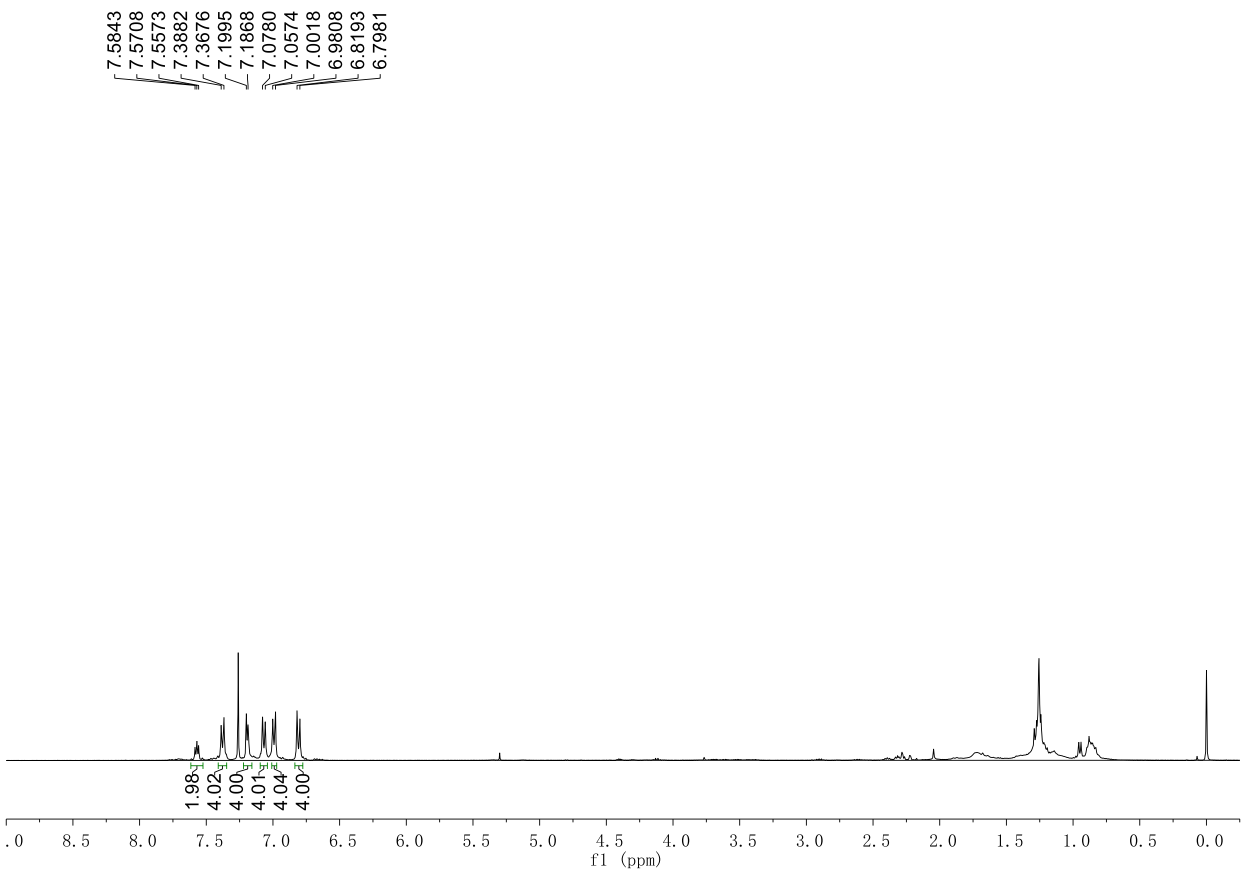

Figure S2. ^{1}H NMR spectra of compound **13**.

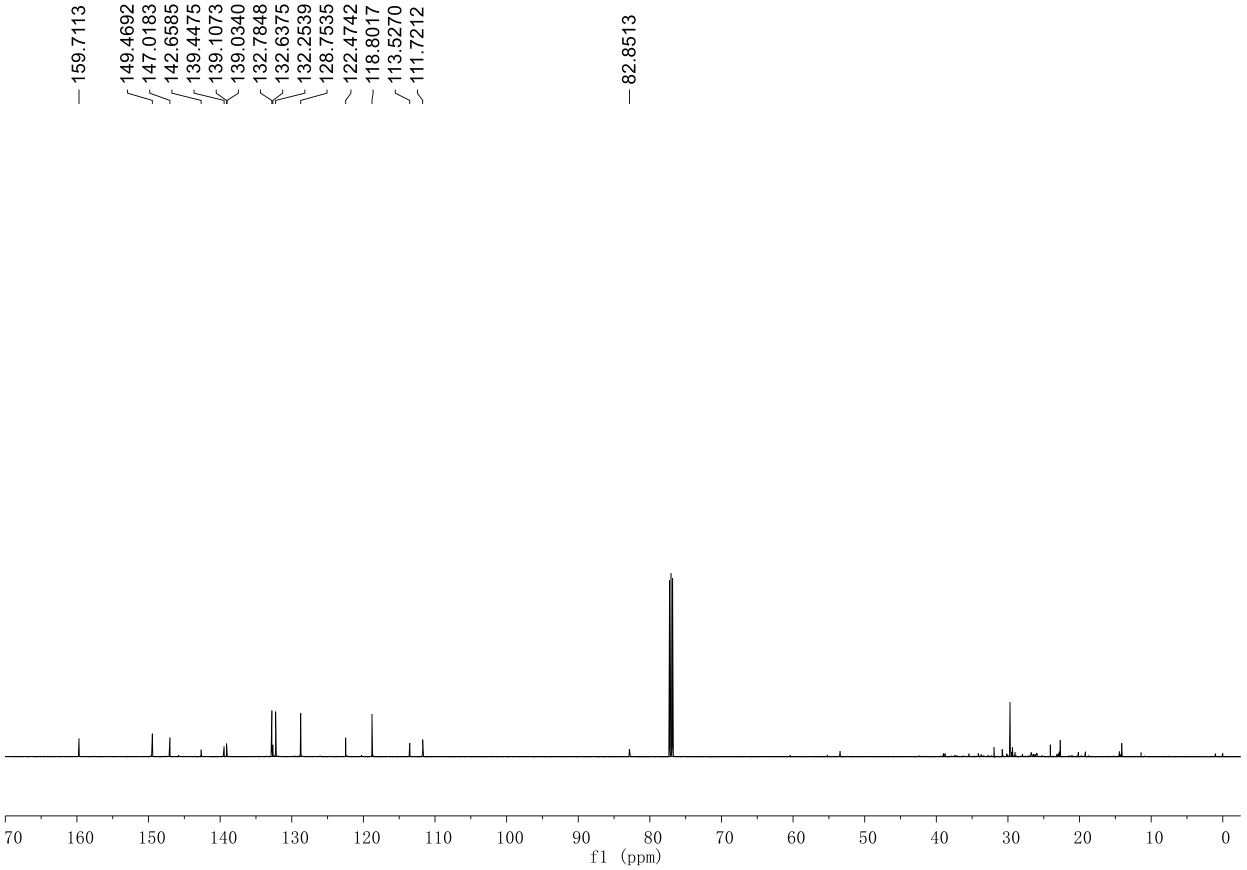

Figure S3. ^{13}C NMR spectra of compound **13**.

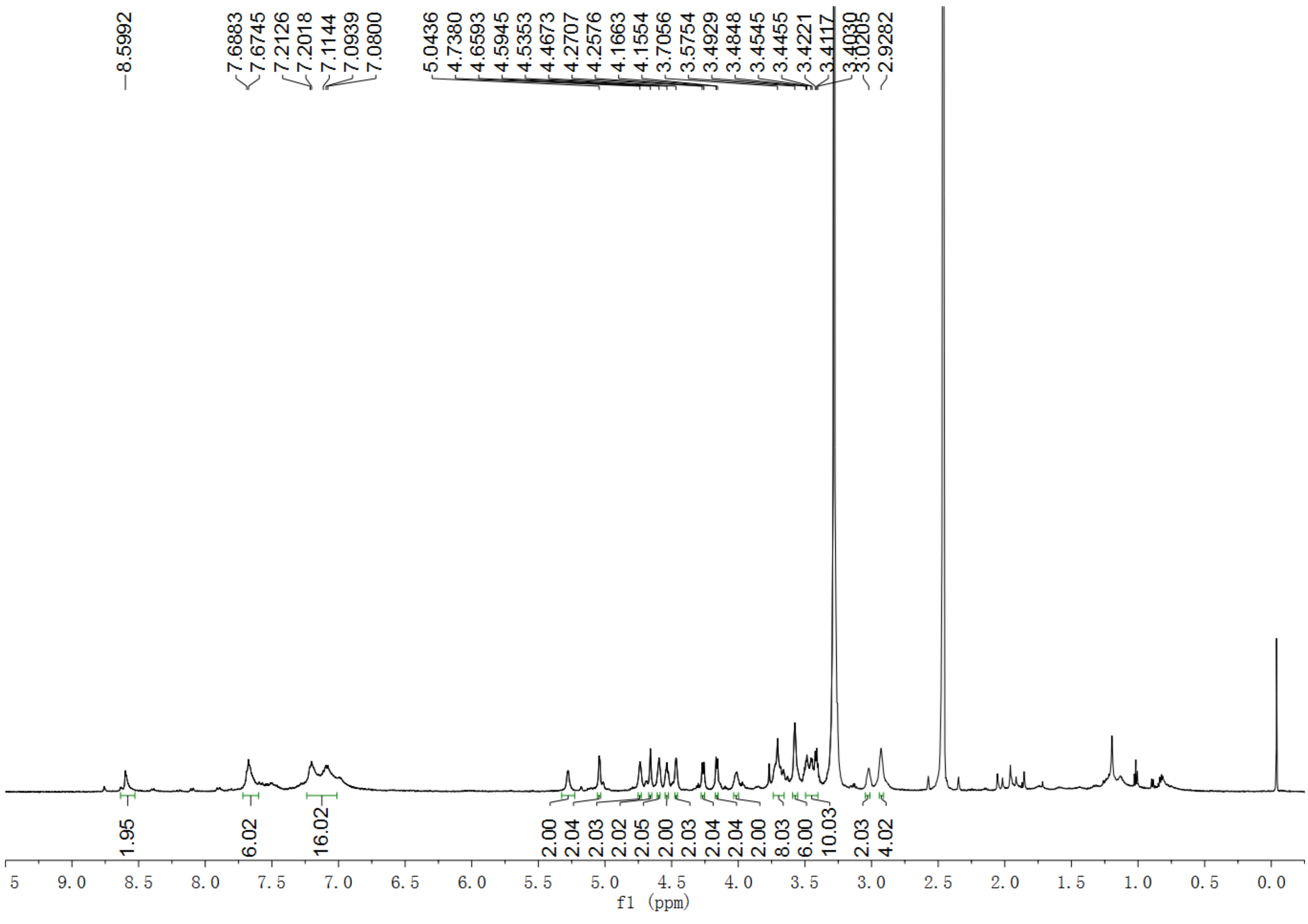

Figure S4. ^{1}H NMR spectra of **TMNL**.

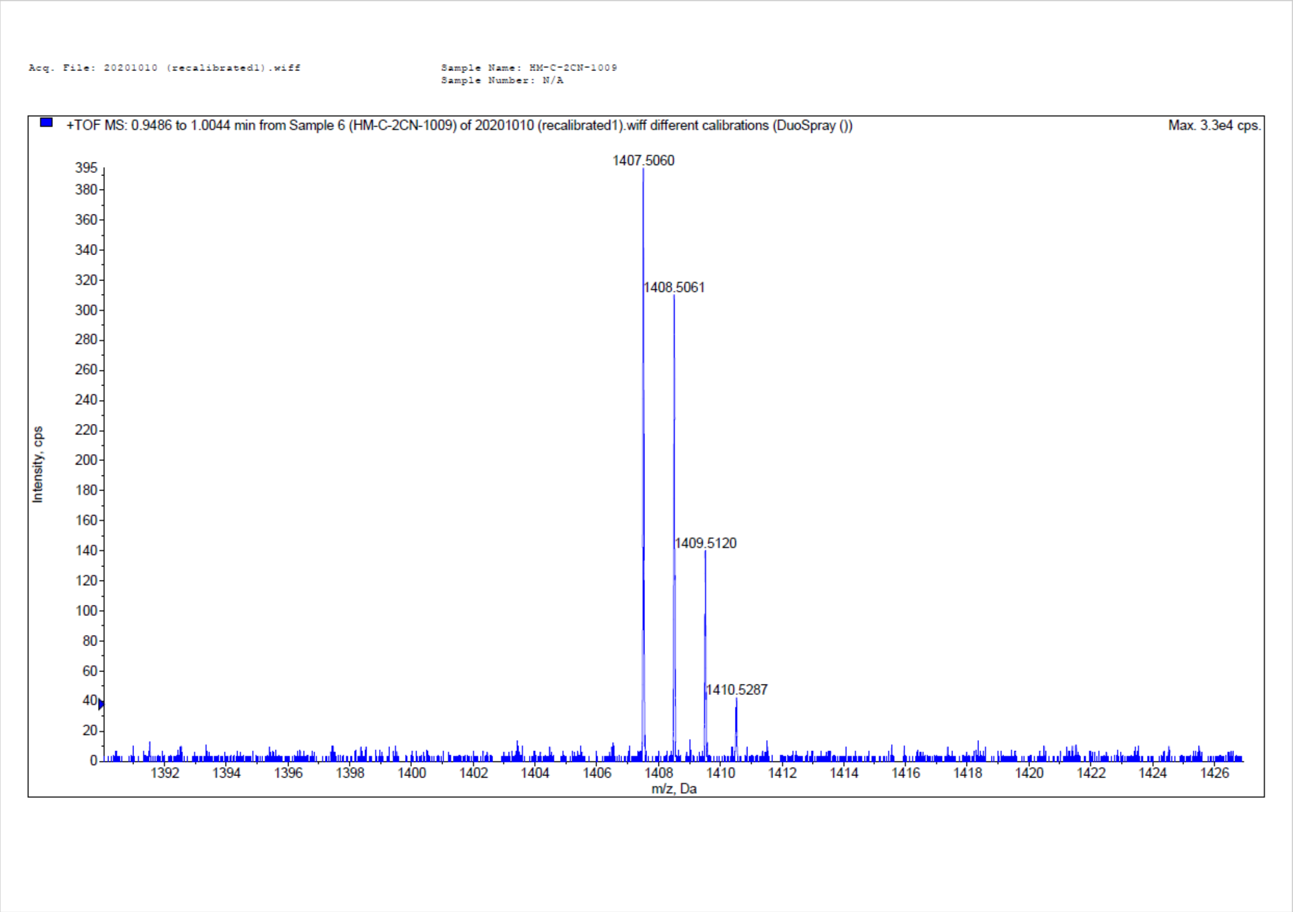

Figure S5. HRMS spectra of **TMNL**.

3 Reference

[1] Yang Y.; Li S.; Zhang Q.; Kuang Y.; Qin A.; Gao M.; Li F.; Tang B.Z. An AIE-active theranostic probe for light-up detection of Aβ aggregates and protection of neuronal cells. *J. Mater. Chem. B* **2019**, *7*, 2434-2441.

[2] Fu W.; Yan C.; Guo Z.; Zhang J.; Zhang H.; Tian H.; Zhu W.H. Rational Design of Near-Infrared Aggregation-Induced-Emission-Active Probes: In Situ Mapping of Amyloid-β Plaques with Ultrasensitivity and High-Fidelity. *J. Am. Chem. Soc.* **2019**, *141*, 3171-3177.

[3] Wang Y.; Qiu Y.; Sun A.; Xiong Y.; Tan H.; Shi Y.; Yu P.; Roy G.; Zhang L.; Yan J. Dual-functional AIE fluorescent probes for imaging β-amyloid plaques and lipid droplets. *Anal. Chim. Acta* **2020**, *1133*, 109-118.

[4] Xu M.; Li R.; Li X.; Lv G.; Li S.; Sun A.; Zhou Y.; Yi T. NIR fluorescent probes with good water-solubility for detection of amyloid beta aggregates in Alzheimer's disease. *J. Mater. Chem. B* **2019**, *7*, 5535-5540.
